# Fabrication of Graphitized Carbon Fibers from Fusible Lignin and Their Application in Supercapacitors

**DOI:** 10.3390/polym15081947

**Published:** 2023-04-19

**Authors:** Linfei Zhou, Xiangyu You, Lingjie Wang, Shijie Qi, Ruichen Wang, Yasumitsu Uraki, Huijie Zhang

**Affiliations:** 1 College of Bioresources Chemical and Materials Engineering, Shaanxi University of Science & Technology, Xi’an 710021, China; linfeizhou@sust.edu.cn (L.Z.);; 2 Research Faculty of Agriculture, Hokkaido University, Sapporo 060-8589, Japan

**Keywords:** lignin, carbon fiber, catalytic graphitization, supercapacitor, electrochemical performance

## Abstract

Lignin-based carbon fibers (LCFs) with graphitized structures decorated on their surfaces were successfully prepared using the simultaneous catalyst loading and chemical stabilization of melt-spun lignin fibers, followed by quick carbonization functionalized as catalytic graphitization. This technique not only enables surficial graphitized LCF preparation at a relatively low temperature of 1200 °C but also avoids additional treatments used in conventional carbon fiber production. The LCFs were then used as electrode materials in a supercapacitor assembly. Electrochemical measurements confirmed that LCF-0.4, a sample with a relatively low specific surface area of 89.9 m^2^ g^−1^, exhibited the best electrochemical properties. The supercapacitor with LCF-0.4 had a specific capacitance of 10.7 F g^−1^ at 0.5 A g^−1^, a power density of 869.5 W kg^−1^, an energy density of 15.7 Wh kg^−1^, and a capacitance retention of 100% after 1500 cycles, even without activation.

## 1. Introduction

Many studies have explored biomaterial production to address environmental deterioration and diminishing oil reserves [1,2,3]. Among biomass resources, as an inexpensive biopolymer with high carbon content and excellent biodegradability, lignin receives considerable interest [4,5]. Despite being the second most common form of biomass on Earth, most lignin is burned for energy recovery, and only 5% of the material is utilized to produce biobased products, causing severe lignin underutilization and wastage [6,7,8,9]. Consequently, novel uses of lignin must be identified to raise its value.

Supercapacitors are a type of energy storage device that can store and release energy quickly and efficiently. They have been used in various applications such as electric vehicles, renewable energy systems, and consumer electronics [10]. Structurally, supercapacitors consist of two electrodes separated with a separator with an electrolyte. The electrode materials play a crucial role in determining the performance of supercapacitors. Carbon fibers (CFs) are a promising electrode material for supercapacitors due to their large surface area, high electrical conductivity, and unique flexibility. However, the major limitation for this application is the high cost of the precursor, pol-yacrylonitrile (PAN; USD 33/kg), which accounts for more than half of CF production costs [11]. In addition, as a carbon source, PAN releases toxic substances during carbonization. Thus, it is necessary to choose a greener carbon source to produce carbon nanofibers.

CFs derived from lignin are promising for the long-term production of high-value commodities as the electrode material in supercapacitors [12,13]. Owing to its high carbon content of over 60%, lignin is a potential alternative to polyacrylonitrile (PAN) for affordable CF production [14,15,16,17,18,19]. The conventional preparation of lignin-based carbon fibers (LCFs) follows these steps: (1) fabricating lignin fibers (LFs) via spinning technology; (2) stabilizing the fibers using chemical or thermal treatment to obtain lignin-based stabilized fibers (LSFs) as CF precursors; (3) carbonization at thousands of degrees Celsius to prepare CFs; and (4) functionalizing the CFs to obtain unique morphological and structural properties, such as a large surface area, appropriate pore size distribution, and graphitic carbon structure [8,20,21,22,23,24,25,26,27,28,29,30,31]. Among these steps, the fiber stabilization process is the most expensive as it takes over 30 h and accounts for a greater proportion of the entire cost of CF production [9]. LCFs with graphitic structures are favored as electrode materials in energy storage devices [32,33,34,35,36]. However, realizing such a structure requires energy-intensive graphitization at 2000–3000 °C [37]. Therefore, investigating high-performance supercapacitors using inexpensive LCFs with unique structures is crucial.

Catalytic graphitization using a metal catalyst is a promising method for transforming an amorphous carbon framework into a graphitic structure. Nickel catalysts have been found to be effective in lowering the graphitization temperature of carbon fibers and are more appealing due to their high abundance on earth [38,39]. To achieve sufficient loading of catalysts, Kubo et al. mixed hardwood acetic acid lignin with a nickelous catalyst and followed this with a series of stirring, drying, and grinding processes [40]. However, this process was unable to keep the carbon fiber form. Choi et al. carried out this catalytic graphitization process using commercial PAN-based CFs [41]. They first removed the sizing material of CFs using acetone solution and then subjected them to catalyst loading and additional heat treatment for several hours at elevated temperatures to enhance catalyst deposition. Therefore, it is still worth investigating the synthesis of low-cost LCFs with graphitized structures as electrode materials.

**Figure 1:**
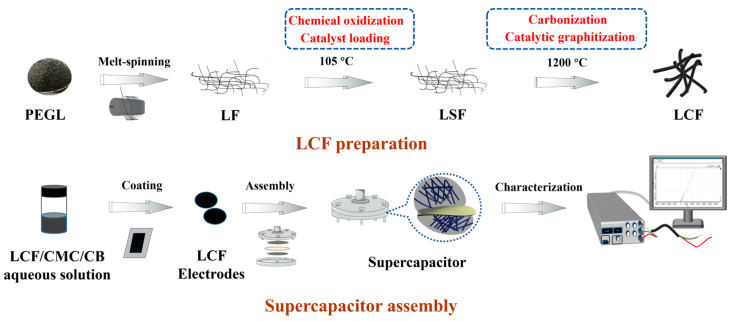
Schematic illustration of LCF preparation and supercapacitor assembly.

Herein, fusible polyethylene glycol lignin (PEGL) fibers were prepared using melt spinning without solvents. Nickelous catalyst loading and melt-spun LF chemical stabilization were then conducted concurrently, followed by fast simultaneous carbonization and catalytic graphitization at 1200 °C. Such a combined LCF preparation process should dramatically decrease the cost. The resulting LCFs with a graphitic structure were used as electrode materials in supercapacitors. The electrochemical performance of the devices was investigated. The scheme of LCF preparation and supercapacitor assembly is presented in Figure 1.

## 2. Materials and Methods

### 2.1. Materials

Poplar wood chips were purchased from Senyi Wood Company (Jinan, China), while polyethylene glycol 400 (PEG 400), HCl, H_2_SO_4_, and NaOH were bought from Damao Chemical Reagent Company (Tianjin, China). Nickel (II) acetylacetonate [Ni(acac)_2_, 95%] was supplied by Aladdin Biochemical Technology Co., Ltd. (Shanghai, China). Carboxymethyl cellulose sodium (CMC) and carbon black (CB) were purchased from Macklin Biochemical Co., Ltd. (Shanghai, China), while triethylmethylammonium tetrafluoroborate (TEMABF_4_) and dimethyl carbonate (PC) were purchased from TCI Development Co., Ltd. (Shanghai, China). All chemicals were used as received.

### 2.2. Melt Spinning of PEGL

PEGL was first isolated from poplar wood chips using organosolv pulping with PEG 400 [42,43,44]. Briefly, 200 g of air-dried wood chips was cooked using 0.3% H_2_SO_4_/PEG 400 at 140 °C for 1.5 h. The liquor-to-wood ratio was 5:1. After the solvolysis reaction, a 0.2 M NaOH aqueous solution was used to extract the lignin components. The extractives were then washed with water and dried to obtain PEGL. Then, PEGL was melt-spun into LFs using a high-pressure melt-spinning apparatus (TL-03; Tongli Co., Ltd., Shenzhen, China). Melt spinning was performed at 150–200 °C under boost pressure between 0.30 and 1.20 MPa. The syringe needle size was 0.44 mm, and the collector rolling speed was 10 rpm.

### 2.3. Preparation of CFs from PEGL

Different amounts of Ni(acac)_2_ were dissolved in 6 M HCl to prepare a homogenous solution. The LFs were then immersed in the solution and kept at 105 °C for 2 h for chemical stabilization to obtain LSFs loaded with Ni(acac)_2_. Then, the LSFs were carbonized in a horizontal tube furnace (GSL-1600X; Hefei Kejing Material Technology Co, Ltd., Hefei, China) using programmed heating under N_2_ stream protection. The program involved 5 °C min^−1^ heating from room temperature to 250 °C, 3 °C min^−1^ heating from 250 °C to 1200 °C with a 1 h dwell at 1200 °C, and a cool down to room temperature to obtain LCFs. These fibers were referred to as LSF-x and LCF-x, where x is the concentration of Ni(acac)_2_/HCl solution ranging from 0 to 0.6 M.

### 2.4. Supercapacitor Assembly

Powdered LCF samples with CB were suspended in a 2 wt% CMC aqueous solution as electrode material slurry. The LCF/CMC/CB weight ratio was maintained at 85:10:5. The slurry was then coated on Al foil to dry. Circular sheets were cut to obtain electrodes with a diameter of 16 mm and an LCF loading of approximately 2.5 mg. Subsequently, the electrodes and cellulosic paper (separator) were both immersed in a 1.2 M TEMABF_4_/PC electrolyte solution and then degassed. Finally, the supercapacitors were assembled by sandwiching the electrodes and separators in a split test cell.

### 2.5. Characterization

#### 2.5.1. Morphological Analysis

The morphologies of the LSF and LCF samples were investigated using field-emission scanning electron microscopy (SEM, S4800, Hitachi, Tokyo, Japan) at an acceleration voltage of 5 kV. The elemental composition on the LCF surfaces was investigated using energy-dispersive X-ray spectroscopy (EDS; Rigaku Corporation, Tokyo, Japan). To quantify the surface areas and porosities of the LSFs and LCFs, we performed N_2_ adsorption/desorption measurements at −196 °C using a chemisorption analyzer (ASAP2460, Micromeritics, San Antonio, TX, USA). The specific surface areas and average pore sizes were calculated using the Brunauer−Emmett−Teller (BET) model. The internal and external specific surface areas were calculated using the t-plot method in a relative pressure range of 0.2–0.5. The pore size distributions were calculated using the non-local density functional theory model.

#### 2.5.2. Thermal Analysis

Thermogravimetry (TG) and differential scanning calorimetry (DSC) were conducted using a dual system (TG–DSC, STA449F3-1053-M, Netzsch, Free State of Bavaria, Germany) from room temperature to 800 °C with a heating rate of 10 °C min^−1^ under a N_2_ atmosphere. The extrapolated onset (T_od_) and endset (T_ed_) decomposition temperatures were obtained from the intersection of the horizontal baseline and tangent at the point of maximum gradient on the TG curves.

#### 2.5.3. Carbonaceous Structure Analysis

Carbon structure identification of the LCFs was conducted using X-ray diffraction (XRD, D8 Advance, Bruker, Karlsruhe, Germany) with CuKα (λ = 1.5406 Å) radiation at 40 kV and 40 mA. XRD patterns were recorded in the 2θ range of 10°–60° at a scan rate of 6° min^−1^. Equations (1) and (2) were used to compute the spacing between the sheets (d_002_) of LCFs [5,12,45,46] and the average crystallite size (L_c_) [5,12]:

(1) $$d_{002}=\frac{\lambda }{2\sin\theta }$$

(2) $$L_{c}=\frac{K\lambda }{\beta \cos\theta }$$

where K = 0.94, θ is the diffraction angle, and β is the peak width at half of the maximum intensity.

Raman spectroscopy with a 532 nm excitation laser (Thermo Scientific DXRxi, Thermo Fisher Scientific, Springfield, MA, USA) was used to characterize the microstructures of the LCF surfaces. The laser power was set to 0.1 mW to avoid overheating the LCF surfaces. Each spectrum was recorded for 300 s. The crystalline sizes (L_a_, in nm) of the LCFs were calculated using Equation (3) [47]:

(3) $$L_{a}=2.4\times 10^{-10}\lambda^{4}R^{-1}$$

where λ is the laser wavelength (nm), and R is the integrated area ratio of the D-band (1350 cm^−1^) to the G-band (1600 cm^−1^) in the Raman spectra.

Fine carbon structural observations were conducted using transmission electron microscopy (TEM, Talos F200s, FEI, Hillsboro, OR, USA) operating at 200 kV with a resolution of 0.14 nm. Before transferring them to the TEM chamber, the powdered LCF samples were dispersed in ethanol, and a droplet of each supernatant was placed on Cu grids.

#### 2.5.4. Electrochemical Performance Measurements

Electrochemical characterization of the assembled two-electrode supercapacitors, including cyclic voltammetry (CV), galvanostatic charge/discharge (GCD), and electrochemical impedance spectroscopy (EIS) analyses, were performed with an electrochemical workstation (Donghua DH7000, Jiangsu Donghua Analytical Instrument Co., Ltd., Suzhou, China). The EIS experiments were conducted between 106 Hz and 1 Hz with an amplitude of 10 mV.

The CV measurements were performed at various scan rates from 0.05 to 0.2 V s^−1^. The gravimetric specific capacitance of a cell (C_CV_) was computed using Equation (4) [48,49,50]:

(4) $$C_{\mathrm{CV}}=\frac{\int i\mathrm{dV}}{m\cdot v\cdot \Delta E}$$

where m is the material loading of the two electrodes, ΔE is the voltage window, i is the response current, and v is the scan rate. In the GCD measurements, a current density of 0.5 A g^−1^ was applied, and the specific capacitance (C_GCD_) was calculated using Equation (5) [48,51]:

(5) $$C_{\mathrm{GCD}}=\frac{i}{m\Delta v/\Delta t}$$

where i is the constant charging and discharging current, Δt represents the time for discharging, and Δv is the operating potential window.

The energy density (E) and power density (P) of the assembled supercapacitors were calculated using Equations (6) and (7), [52,53,54,55,56,57]:

(6) $$E=\frac{C_{\mathrm{GCD}}\cdot \Delta V^{2}}{2}$$

(7) $$P=\frac{3600E}{\Delta t}$$

## 3. Results and Discussion

### 3.1. Morphological and Thermal Analysis

**Figure 2:**
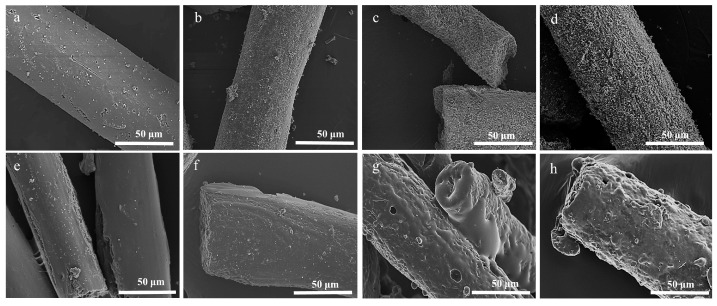
SEM images of (**a**) LSF-0, (**b**) LSF-0.2, (**c**) LSF-0.4, (**d**) LSF-0.6, (**e**) LCF-0, (**f**) LCF-0.2, (**g**) LCF-0.4, and (**h**) LCF-0.6.

**Figure 3:**
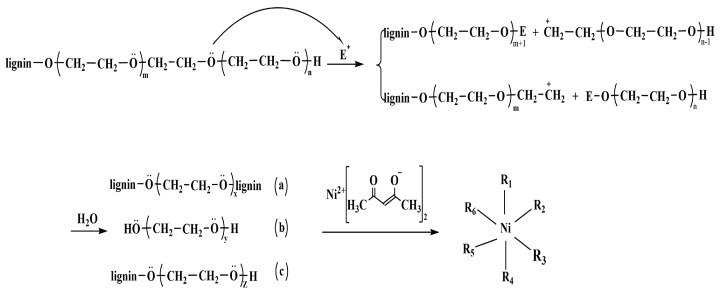
Proposed mechanism of LF chemical stabilization (m, n, x, y, and z ≥ 0; E^+^ represents electrophiles, including H^+^ and Ni^2+^; R_1_, R_2_, R_3_, R_4_, R_5_, and R_6_ represent reaction products a, b, c, acetylacetonate ligands, and other coordinating molecules).

The morphologies and compositions of the LFs, LSFs, and LCFs were observed using SEM and EDS. As shown in Figure S1, the LFs had an average diameter of 158.5 ± 15.9 μm and exhibited smooth surfaces. After chemical stabilization, the fibrous diameters decreased to 53.9 ± 9.6 μm. Pores and splits were generated on the surface of LSF-0 (Figure 2a). With Ni(acac)_2_ addition, the inner and exterior morphologies of the LSFs were clearly altered (Figure 2b–d). These changes can be explained by material interactions. Firstly, ether bond cleavage occurs between the lignin matrix and PEG side chains under concentrated acidic conditions. These residues of low molecular weights wash out easily, forming defects on the LSF surface [58]. Secondly, Ni cations, which are also electrophiles in this system, coordinate different nucleophiles and promote ether bond cleavage, yielding a rough fibrous surface (Figure 3). Additionally, the deposited Ni ions can be a graphitic catalyst in subsequent carbonization [59].

LCF-0 with an average diameter of 64.68 ± 4.6 μm was obtained, as shown in Figure 2e–h. The fiber diameter was reduced to 48.9 ± 5.6 μm after carbonization for LCF-0.4. Notably, a fibrous melt surface was observed for LCF-0.4 and LCF-0.6. With confirmed increased Ni loading via EDS (Figure S2), this melt surface may have been caused by Ni-assisted catalytic graphitization. This graphitization is explained in the following sections.

**Figure 4:**
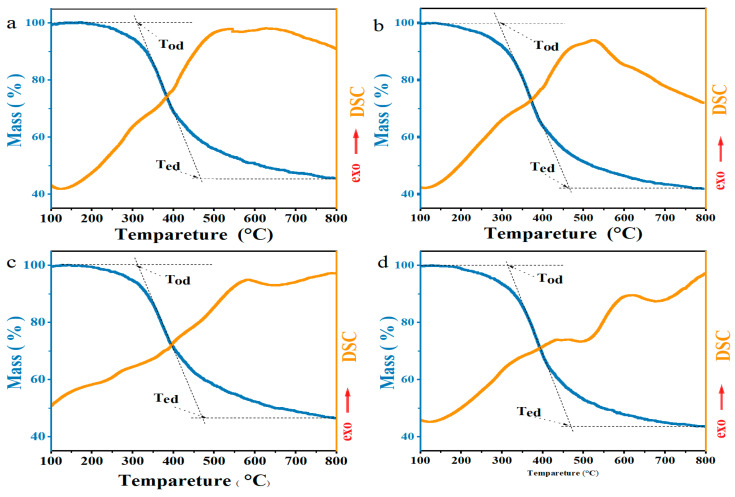
TG and DSC curves of (**a**) LSF-0, (**b**) LSF-0.2, (**c**) LSF-0.4, and (**d**) LSF-0.6.

Simultaneous TG–DSC measurements were performed under a N_2_ atmosphere to investigate the decomposition and melting behavior of the LSFs during carbonization (Figure 4 and Table 1). All the LSFs exhibit one main weight-loss stage in the TG curves, which corresponds to the decomposition of unstable small-molecule components. The onset temperatures of decomposition decreased from 312.7 °C to 289.5 °C with an increasing Ni content. Such decomposition produced a mass loss of 39.4% for LSF-0, which increased to 45.7% for LSF-0.6. This increase proves that Ni assists in ether bond cleavage and small-fragment formation. In the DSC curve above the extrapolated T_ed_, LSF-0 exhibits a broad exothermic region with an exothermic peak at 582.7 °C, which is a typical feature in biomass carbonization [60]. Meanwhile, for LSF-0.2, the DSC heat flow changes from an exothermic to an endothermic reaction at 660.6 °C. This temperature decreases to 491.4 °C and 472.0 °C for LSF-0.4 and LSF-0.6, respectively, indicating the developing ordered carbon structures (graphitic and turbostratic carbons) [60,61,62]. According to the proposed mechanism for catalytic graphitization, the endothermic reaction suggests the dissolution of sp^3^ C atoms in Ni and the precipitation of sp^2^ C in the saturated C/Ni solution, affording the melt surfaces observed in the SEM images.

### 3.2. Structural Determination

**Figure 5:**
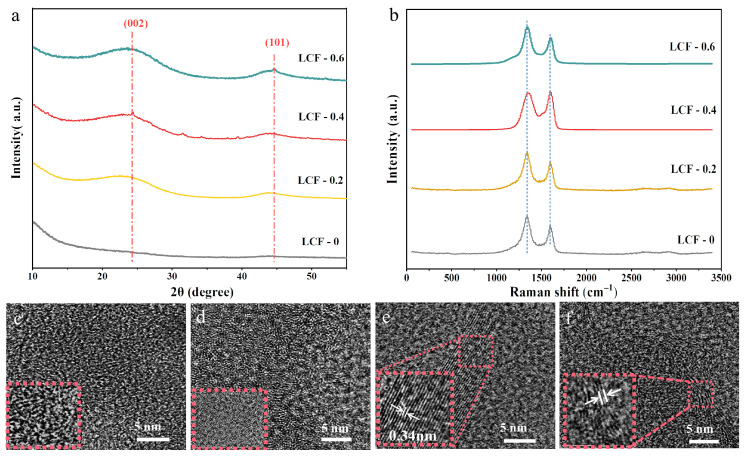
(**a**) Raman spectra, (**b**) XRD patterns, and (**c**–**f**) corresponding TEM images of LCFs with different Ni(acac)_2_ contents: LCF-0, LCF-0.2, LCF-0.4, and LCF-0.6.

To understand the carbon structures of the LCF samples, we performed Raman spectroscopy on the fibers. As shown in Figure 5a, all the LCF spectra had both a D-band at 1350 cm^–1^ and G-band at ~1600 cm^–1^ after deconvolution, where a G-band refers to the in-plane vibration of graphitic carbon atoms, and a D-band corresponds to disordered sp^2^-hybridized graphitic carbon atoms [46]. The integrated area ratio of I_D_/I_G_ (R) can be calculated to gauge the degree of disorder at the surface structures of the LCFs [33]. As shown in Table 1, LCF-0 exhibited the highest R-value of 8.16 among the prepared samples. With an increasing Ni loading, the R-value decreased to 2.57 for LCF-0.4. This sample exhibited the largest La plane size of 7.48 nm among the LCF samples. When the Ni(acac)_2_ amount was further increased, the R and L_a_ values of LCF-0.6 increased to 3.71 and 5.18 nm, respectively, indicating a smaller graphitic region than that of LCF-0.4. This difference may be due to Ni being a graphitization catalyst as well as an etching reagent during chemical stabilization, generating a porous surface on the LSFs. Thus, high Ni concentrations yield aggressive etching, causing discontinuous graphitic carbon regions after carbonization. This phenomenon indicates that biomass graphitization cannot be improved by simply increasing the amount of Ni ions [46,63].

XRD characterization was conducted to elucidate the carbon structure changes from those of the bulk LCF samples. As shown in Figure 5b, no diffraction peaks were observed for LCF-0 in the entire scan range, indicating that only amorphous carbon was generated without Ni(acac)_2_. Meanwhile, broad XRD peaks for the LCF samples at 24° and 44° were identified, which correspond to the (002) and (101) crystal planes, respectively. This is because Ni ions could only be adsorbed on the surfaces of the LSFs during chemical stabilization and could not access the insides. The average Lc values for LCF-0.2, LCF-0.4, and LCF-0.6, based on the (002) peaks, were 0.26 nm, 0.41 nm, and 0.39 nm, respectively (Table 2).

High-resolution TEM clearly shows nanostructures in the LCFs (Figure 5). Consistent with the XRD and Raman spectroscopy results, ordered microstructures were difficult to find in LCF-0 and LCF-0.2 owing to low Ni contents on the fibrous surfaces. For LCF-0.4 and LCF-0.6, distinct lattice fringes embedded in the amorphous regions were identified. LCF-0.4 exhibited large ordered layers. The measured spacing between the crystal layers was 0.34 nm, proving Ni-assisted graphitic structure generation. Thus, the LCFs comprised mainly amorphous carbon in the bulk covered with crystallized carbon layers on the surface. Such a structure would be beneficial for electrolyte ion transport and accumulation in supercapacitors.

The N_2_ adsorption/desorption curves (Figure S3) exhibit typical type-I behavior. The specific surface areas and pore information for the LCFs are listed in Table 3. The specific surface area of LCF-0 was 350.95 m^2^ g^−1^, with 316.67 m^2^ g^−1^ from micro/mesopores and 34.28 m^2^ g^−1^ from macropores, according to the t-plot method. The specific surface area decreased with the Ni(acac)_2_ content and reached a minimum value of 89.92 m^2^ g^−1^ for LCF-0.4. Moreover, the total volumes and internal/external surface areas exhibited a similar trend. This can be explained by Ni-induced melting on the fiber surfaces, which filled up several pores and decreased the specific surface areas. Thus, the total pore volume decreased from 0.13 m^3^ g^−1^ for LCF-0 to 0.03 m^3^ g^−1^ for LCF-0.4. Interestingly, the surface area and total pore volume increased to 269.56 m^2^ g^−1^ and 0.11 m^3^ g^−1^ for LCF-0.6, respectively. This may be due to the influence of Ni ions on etching rather than filling the surfaces at high Ni concentrations in the Ni-treated LCF samples. This feature is expected to influence supercapacitor performance, as discussed in the next section [19,64,65,66].

### 3.3. Electrochemical Performance of the LCF-Based Supercapacitors

**Figure 6:**
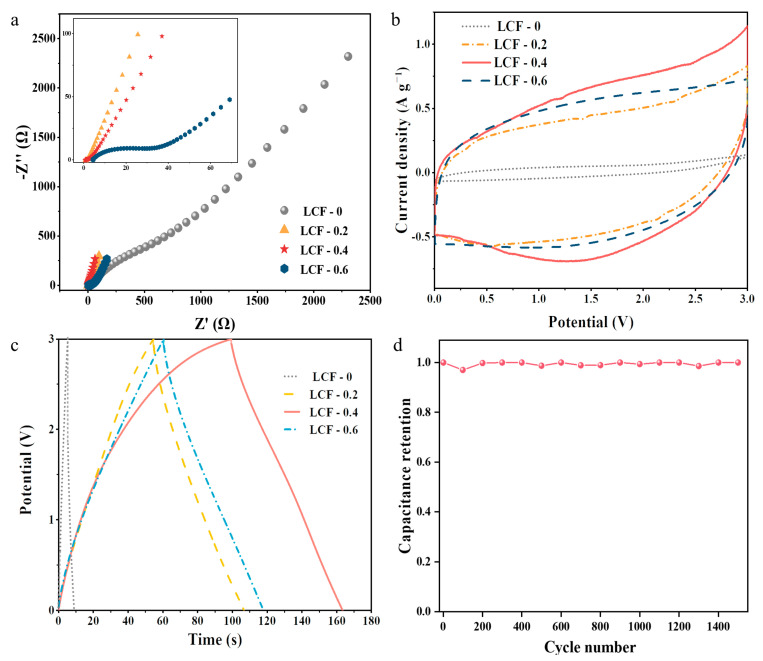
Electrochemical performance of the LCF-based supercapacitors. (**a**) Nyquist plots and (**b**) CV curves of the LCF electrodes at a scan rate of 0.05 V s^−1^. (**c**) GCD curves at a current density of 0.5 A g^−1^. (**d**) Capacitance retention at 1500 cycles.

EIS, CV, and GCD experiments using a two-electrode system with a 1.2 M TEMABF_4_/PC electrolyte were performed to determine the electrochemical performance of the LCF-based electrodes. The organic electrolyte is not corrosive to the casing and has a higher working voltage than the aqueous electrolyte. As shown in Figure 6a, the Nyquist plots in the high-frequency region show the electrode resistance (R_e_) of the supercapacitors at the *x*-axis intercept, which indicates the resistances of the electrode materials and their contacts. The equivalent series resistance (R_s_) was obtained from the intersection of the straight line in the low-frequency region with Z_re_ axis. Rs is the sum of the electrode and electrolyte resistances [67,68]. Without graphitic structures on its surface, LCF-0 had very high R_e_ (39.7 Ω) and R_s_ (43.6 Ω) values even when CB was added to the electrode recipe (Table 4). When Ni(acac)_2_ was used in chemical thermostabilization, the Re and Rs decreased remarkably and achieved the lowest values of 0.9 and 1.2 Ω for LCF-0.4, respectively. The low resistances suggest that a low-resistance network was established on the surface of LCF-0.4 owing to Ni catalytic graphitization. Additionally, the straight line in the low-frequency region for LCF-0.4 exhibits a higher slope among the LCF samples, indicating the rapid diffusion of electrolyte ions during the charge–discharge process.

The CV curves of the supercapacitors with the LCFs exhibited quasi-rectangular shapes within the range of 0–3 V, indicating typical supercapacitor behavior (Figure 6b). Even though LCF-0 had a large surface area and well-developed porous structure, the capacitance of the assembled supercapacitor was only 1.3 F g^−1^ at a scan rate of 0.05 V s^−1^. This value increased considerably for the Ni-catalyzed LCF electrodes, and a high capacitance of up to 22.0 F g^−1^ was obtained for LCF-0.4.

**Figure 7:**
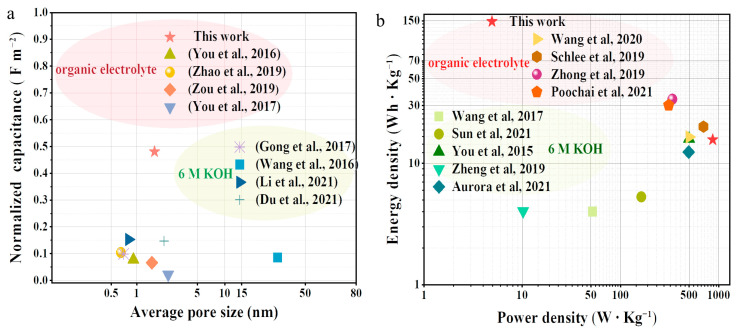
(**a**) Plots for specific capacitance normalized by surface area vs. average pore size [33,36,44,48,57,64,71,78]. (**b**) Ragone plots for the LCFs [3,34,47,50,55,70,72,73,74].

GCD measurements were conducted to further evaluate the electrochemical performance of the LCF electrodes (Figure 6c). A very short discharge time of 3 s was observed when LCF-0 was used as the electrode. The discharge time extended to 65 s and a specific capacitance of 10.7 F g^−1^ was obtained when graphitic LCF electrodes at the same current density (0.5 A g^−1^) were employed. These findings are consistent with the CV results. It can also be observed that the IR drop for LCF-0.4 displayed smaller values than that of the other three electrode materials, which is consistent with the internal resistance retrieved from the sum of the electrode and bulk electrolyte resistances in the EIS results. Moreover, LCF-0.4 exhibited an excellent electrochemical stability of 100% after 1500 cycles. Notably, the normalized capacitance of the LCF-0.4 electrode outperformed those of most reported biomass-derived supercapacitors at 0.5 A g^−1^ (Figure 7a) [69,70,71]. Thus, the assembled supercapacitor using LCF-0.4 exhibited the best performance. It had an energy density of 15.7 Wh kg^−1^ and a power density of 869.5 W kg^−1^, which are comparable to those of previously reported biomass-derived supercapacitors (Figure 7b) [3,72,73,74,75,76,77]. Thus, we infer that the Ni-catalyzed graphitization-induced conductive network had more influence on electrolyte ion accumulation than on surface area and pore size distribution, producing high electrochemical performance.

## 4. Conclusions

In summary, we demonstrated a simple preparation procedure for LCFs involving loading the catalyst during chemical stabilization and combining graphitization and carbonization. The resulting LCFs mainly comprise bulk amorphous carbon covered with graphitic carbon layers on the surface. Although LCF-0.4 exhibited a low specific surface area of 89.9 m^2^ g^−1^ and a relatively small total pore volume of 0.03 m^3^ g^−1^, the assembled supercapacitor using this material exhibited excellent electrical properties even without activation. The supercapacitor had a capacitance of 10.7 F g^−1^ at 0.5 A g^−1^, a power density of 869.5 W kg^−1^ at an energy density of 15.7 Wh kg^−1^, and no capacitance loss after 1500 cycles. Therefore, transforming melt-spun LFs into LCFs with graphitic surfaces is an inexpensive and effective strategy to prepare high-performance electrode materials that are promising for energy storage and biosensors.

## Figures and Tables

**Table 1:** Decomposition temperatures and mass loss analysis of the LSF samples.

Sample	Extrapolated Onset Decomposition Temperature (T_od_, °C)	Extrapolated Endset Decomposition Temperature (T_ed_, °C)	Mass Loss at Decomposition (%)	Mass Loss at Carbonization and Graphitization (%)	Total Mass Loss (%)
LSF-0	312.7	471.7	39.4	14.1	53.5
LSF-0.2	309.1	468.4	41.2	13.3	54.5
LSF-0.4	308.4	467.9	43.9	12.5	56.4
LSF-0.6	289.5	463.5	45.7	12.4	58.1

**Table 2:** Calculated Raman spectroscopy and XRD results for the LCFs.

Sample	Raman		XRD	
	R = I_D_/I_G_	L_a_ (nm)	L_c_ (nm)	d_002_ (nm)
LCF-0	8.16	2.36	–	–
LCF-0.2	3.74	5.14	0.26	0.37
LCF-0.4	2.57	7.48	0.41	0.35
LCF-0.6	3.71	5.18	0.39	0.37

**Table 3:** Textural properties of the LCFs with varying Ni(acac)_2_ contents.

Samples	BET ^a^ (m^2^ g^−1^)	W_p_^b^ (nm)	V_total_^c^ (m^3^ g^−1^)	S_internal_^d^ (m^2^ g^−1^)	S_external_^e^ (m^2^ g^−1^)
LCF-0	350.95	1.62	0.13	316.67	34.28
LCF-0.2	103.52	1.65	0.04	93.96	9.57
LCF-0.4	89.92	1.61	0.03	83.74	6.19
LCF-0.6	269.56	1.67	0.11	234.48	35.08

^a^ S_BET_: specific surface area computed using the BET model. ^b^ W_p_: adsorption average pore width. ^c^ V_total_: total pore volume. ^d^ S_internal_: surface area of micropores and mesopores using the t-plot method. ^e^ S_external_: surface area of macropores using the t-plot method.

**Table 4:** Summarized supercapacitor performance using different LCF electrodes.

Sample	C_CV_ ^a^ (F g^−1^)	C_GCD_ ^b^ (F g^−1^)	R_s_ ^c^ (Ω)	IR ^d^ (V)	E ^e^ (Wh kg^−1^)	P ^f^ (W kg^−1^)
LCF-0	1.3	0.6	6.3	0.10	0.8	767.9
LCF-0.2	16.3	8.7	2.0	0.07	11.7	811.6
LCF-0.4	22.0	10.7	1.2	0.05	15.7	869.5
LCF-0.6	18.9	9.6	21.0	0.08	13.2	829.3

^a^ C_CV_: gravimetric specific capacitance calculated from CV profiles. ^b^ C_GCD_: specific capacitance calculated from GCD profiles. ^c^ Rs: equivalent series resistance from Nyquist plots. ^d^ IR: voltage drop. ^e^ E: energy density. ^f^ P: power density.

## Supplementary Materials

The following supporting information can be downloaded at https://www.mdpi.com/article/10.3390/polym15081947/s1: Figure S1. SEM images of LFs; Figure S2. EDS mapping of LCFs, including SEM image of (a) LCF-0, (e) LCF-0.2, (i) LCF-0.4, and (m) LCF-0.6; Figure S3. N2 adsorption/desorption isotherms; Figure S4. (a) CV for LCF-0.4 with different scan rate; (b) GCD curves for LCF-0.4 at different current density.
