# Supplementary material for: Fabrication of Graphitized Carbon Fibers from Fusible Lignin and Their Application in Supercapacitors
Source: Polymers (Basel). 2023 Apr 19;15(8):1947. doi: 10.3390/polym15081947 (PMC10142849; doi:10.3390/polym15081947)
Supplement: Supplementary file 1 [file polymers-15-01947-s001.zip › polymers-2316657-supplementary.pdf]

polymers

Supplementary material

Fabrication of Graphitized Carbon Fibers from Fusible Lignin and Their Application in Supercapacitors

Linfei Zhou [1], Xiangyu You [1,*], Lingjie Wang [1], Shijie Qi [1], Ruichen Wang [1], Yasumitsu Uraki [2] and Huijie Zhang [1,*]

1 College of Bioresources Chemical and Materials Engineering, Shaanxi University of Science & Technology, Xi'an 710021, China; linfeizhou@sust.edu.cn (L.Z.); 13177274057@163.com (L.W.); 210111015@sust.edu.cn (S.Q.); wang.ruichen@sust.edu.cn (R.W.)
2 Research Faculty of Agriculture, Hokkaido University, Sapporo 060-8589, Japan; uraki@for.agr.hokudai.ac.jp
* Correspondence: xyyou@sust.edu.cn (X.Y.); hjzhang@sust.edu.cn (H.Z.)

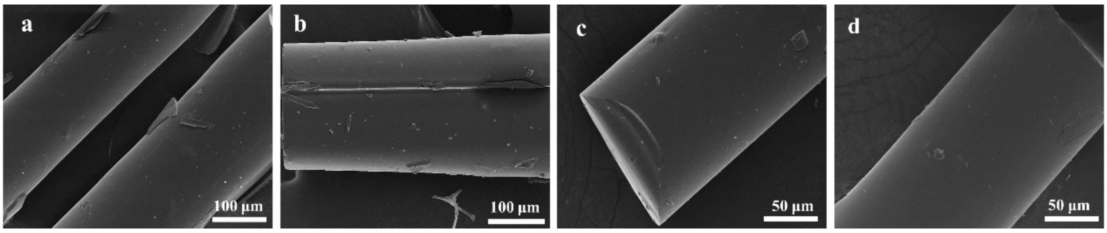

Figure S1. SEM images of LFs.

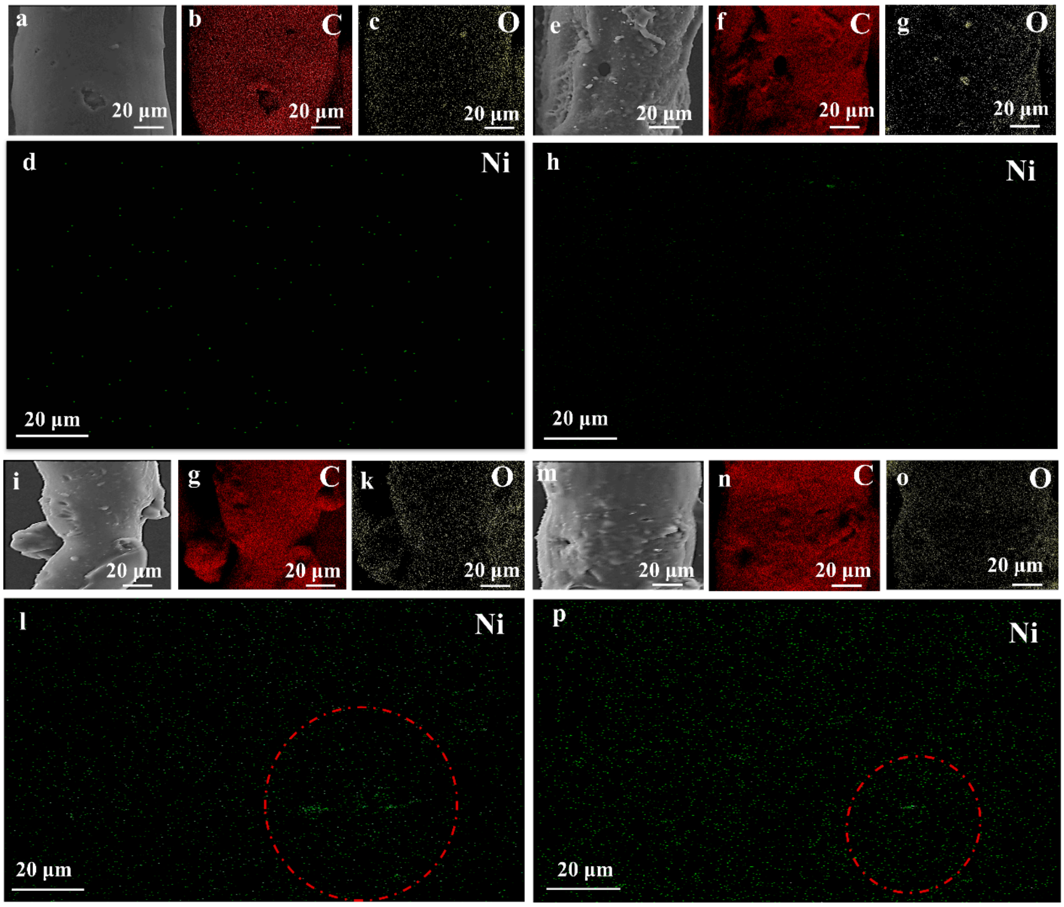

Figure S2. EDS mapping of LCFs, including SEM image of (a) LCF-0, (e) LCF-0.2, (i) LCF-0.4, and (m) LCF-0.6.

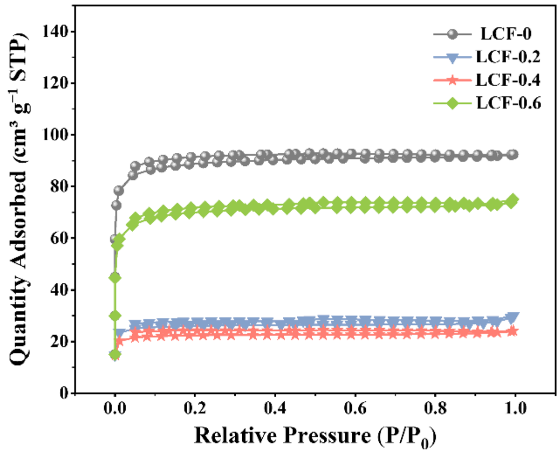

Figure S3. N_2 adsorption/desorption isotherms.

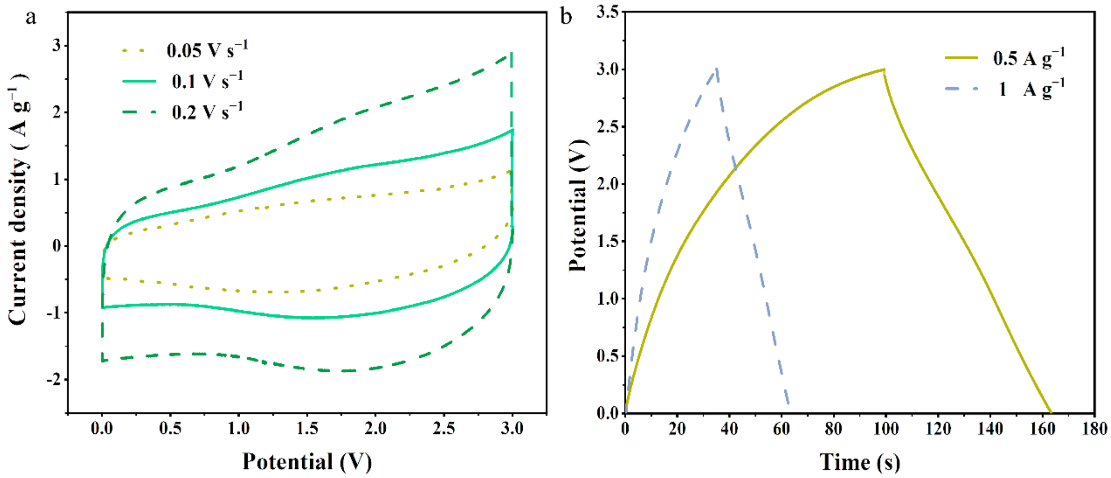

Figure S4. (a) CV for LCF-0.4 with different scan rate; (b) GCD curves for LCF-0.4 at different current density.
